# Concordance analysis of left ventricular mass by transthoracic echocardiography versus 64-slice multidetector computed tomography

**DOI:** 10.1186/cc9450

**Published:** 2011-03-11

**Authors:** JJ Jimenez, JL Iribarren, J Lacalzada, A Barragan, M Brouard, I Laynez

**Affiliations:** 1 Hospital Universitario de Canarias, La Laguna, Spain

## Introduction

Left ventricular mass (LVM) is considered an independent cardiovascular risk factor. Today we have new cardiac imaging methods for its calculation, which are incorporated into the already established classic. The aim of the study was to assess a comprehensive analysis of the correlation of LVM between two different diagnostic techniques, transthoracic echocardiography (TTE) and 64-slice multidetector computed tomography (MDCT).

## Methods

A prospective cohort of 102 patients' LVM was quantified by TTE and MDCT in a row and blind study. We used the following test: intraclass correlation coefficient absolute agreement (ICCA) as a mixed model, concordance correlation coefficient of Lin (CCCL) to evaluate the accuracy, Passing-Bablock regression (PBR) to detect systematic errors and finally the range of Bland-Altman agreement.

## Results

There were 57 (55.8%) males, mean age 65 ± 13 years. ICCA was 0.67 (95% CI: 0.30 to 0.84), *P *< 0.001; the CCCL was 0.67. The PBR (*Y *= *A *+ B * *X*) was: *A *= -29 (95% CI: -170 to 64), *B *= 0.70 (95% CI: 0.51 to 0.98). The range of agreement of Bland-Altman showed a mean of *X *(TTE) - *Y *(MDCT) = -37.8 (95% CI: -47 to 72) g, there were two cases below the lower limit.

## Conclusions

Both methods show a level of consistency and acceptable accuracy, showing no systematic error constant rate (interval *A *contains 0) but there seems to be a discrete proportional error (interval *B *does not contain 1). As shown, the Bland-Altman range seems to slightly overestimate the TTE value against the MDCT, probably related to the quality of the echocardiography window.

